# Nighttime ambulatory pulse pressure predicts cardiovascular and all‐cause mortality among middle‐aged participants in the 21‐year follow‐up

**DOI:** 10.1111/jch.14317

**Published:** 2021-07-03

**Authors:** Päivi A. Lempiäinen, Antti Ylitalo, Heikki Huikuri, Y. Antero Kesäniemi, Olavi H. Ukkola

**Affiliations:** ^1^ Research Unit of Internal Medicine Medical Research Center Oulu Oulu University Hospital University of Oulu Oulu Finland; ^2^ Heart Center Turku University Hospital and University of Turku Turku Finland

**Keywords:** ambulatory pulse pressure, all‐cause mortality, cardiovascular mortality, follow‐up, nighttime pulse pressure

## Abstract

Office pulse pressure (PP) is a predictor for cardiovascular (CV) events and mortality. Our aim was to evaluate ambulatory PP as a long‐term risk factor in a random cohort of middle‐aged participants. The Opera study took place in years 1991–1993, with a 24‐h ambulatory blood pressure measurement (ABPM) performed to 900 participants. The end‐points were non‐fatal and fatal CV events, and deaths of all‐causes. Follow‐up period, until the first event or until the end of the year 2014, was 21.1 years (mean). Of 900 participants, 22.6% died (29.6% of men/15.6% of women, *p*<.001). A CV event was experienced by 208 participants (23.1%), 68.3% of them were male (*p*<.001). High nighttime ambulatory PP predicted independently CV mortality (hazard ratio [HR] 2.60; 95% confidence interval [CI 95%] 1.08–6.31, *p*=.034) and all‐cause mortality in the whole population (HR 1.72; Cl 95% 1.06–2.78, *p*=.028). In males, both 24‐h PP and nighttime PP associated with CV mortality and all‐cause mortality (24‐h PP HR for CV mortality 2.98; CI 95% 1.11–8.04, *p*=.031 and all‐cause mortality HR 2.40; CI 95% 1.32–4.37, *p*=.004). Accordingly, nighttime PP; HR for CV mortality 3.13; CI 95% 1.14–8.56, *p*=.026, and for all‐cause mortality HR 2.26; CI 95% 1.29–3.96, *p*=.004. Cox regression analyses were adjusted by sex, CV risk factors, and appropriate ambulatory mean systolic BP. In our study, high ambulatory nighttime PP was detected as a long‐term risk factor for CV and all‐cause mortality in middle‐aged individuals.

## 1 INTRODUCTION

Pulse pressure (PP) is defined as the difference between systolic blood pressure (SBP) and diastolic blood pressure (DBP) and it is considered as a surrogate marker for arterial stiffening (viite). PP increases with ageing, as SBP increases, and DBP decreases.^1^, ^2^ High office PP is a known independent risk factor for cardiovascular (CV) events and CV mortality,^2^, ^3^, ^4^, ^5^, ^6^, ^7^ especially in individuals over 60 years of age,^2^, ^7^ but also in the middle‐aged participants.^4^, ^5^, ^6^, ^8^ Increased office PP is also associated with all‐cause mortality in a middle‐aged male population,^4^ and in young adults.^9^ A rising trend in PP over a period of time was discovered to increase the risk for all‐cause mortality in a large population study with a wide age group.^10^ In the very elderly however, PP may not be a CV risk factor.^11^

There is evidence that ambulatory PP could be an even better method in predicting the risk of CV events and mortality than office PP,^8^, ^12^ or ambulatory systolic blood pressure (BP).^8^, ^13^ Increased ambulatory PP has been found as a CV risk factor in different specific patient groups: it predicts CV events in patients with peripheral artery disease,^14^ CV mortality in hemodialysis patients,^15^ and also a variety of adverse CV outcomes and all‐cause mortality in participants with hypertension.^16^ In a population study, daytime and 24‐h PP had a predictive value in the general population, whereas the nighttime PP was a risk factor for men.^17^ In a large study consisting of 11 different populations, all individuals within the highest 24‐h PP distribution had an elevated risk for CV events, and those over 60 years had the higher all‐cause mortality than those below.^18^ In some studies, however, there has been controversy over significance of ambulatory PP as a predictor for CV and all‐cause mortality.^19^

In the present study, our aim was to investigate ambulatory PP (24‐h, daytime, and nighttime) as a predictor for long‐term cardiovascular events and all‐cause mortality in a middle‐aged population as a whole, and also separately in both sexes, because sex differences have been reported earlier.^17^, ^20^ To our knowledge, population based studies targeting ambulatory PP as a predictive factor for CV events or mortality with such a long follow‐up period as ours do not exist.

## 2 METHODS

### 2.1 Study population

**Figure 1:**
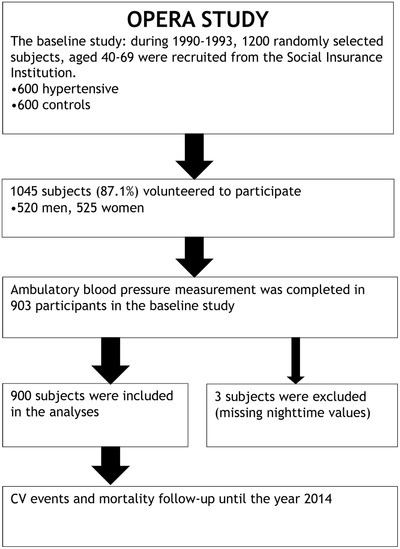
Flow chart of the OPERA Study ‐ Oulu Project Elucidating Risk of Atherosclerosis.

This study is part of the OPERA (Oulu Project Elucidating Risk of Atherosclerosis) project, a prospective, population‐based cohort study designed to evaluate the risk factors of atherosclerotic cardiovascular diseases. The details have been published earlier.^21^ In short, a cohort of 600 hypertensive (300 women and 300 men) inhabitants of city of Oulu (106,500 inhabitants) in Northern Finland was randomly selected from the register for the reimbursement of antihypertensive medication maintained by the Social Insurance Institution. An age‐ and sex‐matched control cohort consisting of 600 participants was randomly sampled from the social insurance register of the area. Study participants were 40–62 years of age at the time of recruitment. Out of the 1200 invited, 1045 individuals (87.1%, 525 women, 520 men) participated in the baseline study which was carried out between years 1990 and 1993, and ABPM was recorded in 903 participants. In our analyses we included those, whose ABPM was available, a total of 900 participants. Three participants were excluded because of missing nighttime values of ABPM (Figure 1). Further details of the baseline study can be found elsewhere.^22^ Our study was approved by the Ethics Committee of the Faculty of Medicine, University of Oulu, and was conducted by the principles of the Declaration of Helsinki. All study participants gave an informed consent.

### 2.2 Baseline study

The participants of the study underwent a clinical examination, including height and weight measurements. A questionnaire covering the past medical history, medication use, alcohol consumption, and smoking habits was completed by two trained nurses. The laboratory tests, including a 2‐h oral glucose tolerance test, were obtained after an overnight fast, and analyzed in the Central Laboratory of the Oulu University Hospital.^21^ The estimated glomerular filtration rate (eGFR) was calculated by using CKD‐EPI equation.^23^ Body mass index was calculated as weight (kg) divided by height squared (m). Type 2 diabetes was determined by the WHO criteria^24^: diabetes was diagnosed if fasting plasma glucose was ≥7.0 mmol/L and/or 2‐h plasma glucose was ≥11.1 mmol/L in 2‐h oral glucose tolerance test, or if a person was on medication for diabetes. Hypertension was defined as blood pressure over 140/90 mmHg or current use of antihypertensive medication. A comprehensive report of the baseline study has been published earlier.^21^

### 2.3 Blood pressure measurements

The office BP was measured by a specially trained registered nurse, using an appropriately fitted cuff size and automatic oscillometric device (Dinamap Procare 100, Criticon, Tampa, FL, USA)^25^ when the participants were seated for the minimum of 5 min. BP was then measured at 1 min intervals three times. The mean of the second and the third measurement was used for the analyses.

ABPM was recorded by a noninvasive fully automatic SpaceLabs90207 oscillometric unit (SpaceLabs Inc., Redmond, WA, USA). The measurements were taken every 15 min from 04:00 AM to 12:00 PM and every 20 min between 12:00 PM and 04:00 AM. The British Hypertension Society and the US Association for the Advancement of Medical Instrumentation have previously confirmed the accuracy and reproducibility of the BP readings acquired with this device. The proper positioning of the cuff was ensured in each individual by means of the similarity (difference <5 mmHg) between four SpaceLabs BP measurements and four auscultatory readings using a Y‐connector and the patients were instructed to relax their arm during the measurement. Values were automatically excluded from the analysis if systolic BP (SBP) was less than 70 or more than 250 mmHg, diastolic BP (DBP) less than 40 or more than 150 mmHg, and heart rate less than 40 or more than 150 beats per minute. Less than 3% of the BP readings were rejected as artifacts based on these criteria.^26^ Three recordings were excluded because of missing nighttime variables. Pulse pressure was calculated as a difference between systolic BP and diastolic BP.

### 2.4 Follow‐up of cardiovascular events and mortality

Follow‐up time was defined as the time from the date of the baseline examination to the first end‐point event (CV event or death), or, if event free, until December 31, 2014. The mean follow‐up time was 21.1 years (median 22.5 years, range 18.0–23.2 years).

The primary end‐point was CV event—a major coronary heart disease (CHD) event or stroke, non‐fatal or fatal, whichever occurred first. The secondary end‐point was death of all‐causes. Information on events leading to hospitalization was obtained from the Care register for health care of the Finnish institute for health and welfare. Data on fatal outcome and causes of death were obtained from death certificates from the Archive of death certificates of Statistics Finland. Each study participant was identified and followed with a personal social security number. The diagnoses were classified according to the International Classification of Diseases, Ninth Revision (ICD‐9) before 1994 and the Tenth Revision (ICD‐10) thereafter. CHD was based on the following diagnosis: I20, I21, I22 (ICD‐10) / 410, 4110 (ICD‐9) as the main diagnosis, and I21, I22 (ICD‐10) / 410 (ICD‐9) as a first or second side diagnosis or third diagnosis (ICD‐9 only), or coronary artery bypass grafting, or coronary angioplasty. CHD as a cause of death included I20–I25, I46, R96, R98 (ICD‐10) / 410–414, 798 (not 7980A) (ICD‐9) as the underlying or immediate cause of death and I21 or I22 (ICD‐10) / 410 (ICD‐9) as the first to the third contributing cause of death. Stroke as an end‐point included I61, I63 (not I63.6), I64 (ICD‐10), and 431, 4330A, 4331A, 4339A, 4340A, 4341A, 4349A, 436 (ICD‐9) as the main diagnosis or as a side diagnosis, or as a cause of immediate or contributing cause of death.

### 2.5 Statistical methods

Data analyses were performed with IBM SPSS Statistics for Windows (IBM Corp. Released 2017. IBM SPSS Statistics for Windows, Version 25.0 Armonk, NY: IBM Corp.). Data are expressed as mean ± standard deviation for continuous variables, or median with 25th and 75th quartiles for skewed variables. Continuous variables were tested for difference between the groups with Student's *t* test or with Mann–Whitney's test, when appropriate. Pearson's Chi‐square test was used to test differences between categorical variables. Log transformation was made for fasting insulin and triglycerides. Hazard curves were estimated by using the Kaplan–Meier method and tested by log‐rank test. We used Cox regression analysis to estimate the pulse pressure tertiles as a predictor for cardiovascular events and all‐cause mortality in multivariate models adjusted by variables with significance in univariate analyses. 24‐h PP range was 32–44 mmHg in the lowest tertile (T1), 45–51 mmHg in the middle (T2), and 52–89 mmHg in the highest tertile (T3). Daytime PP was 24–45 mmHg in T1, 46–52 mmHg in T2, and 53–93 mmHg in T3. Nighttime PP range was 24–42 mmHg (T1), 43–48 mmHg (T2), and 49–88 mmHg (T3). All of the fully‐adjusted regression analyses in this study also included the corresponding ambulatory mean SBP as a cofactor: either 24‐h SBP for models assessing 24‐h PP, or daytime SBP for models with daytime PP, or nighttime SBP for models evaluating nighttime PP. *p* values <.05 were considered as statistically significant.

## 3 RESULTS

Originally 1045 participants attended the OPERA study. All those (n=900, 50.4% women, and 49.6% men) who underwent a qualified 24‐h ABPM recording in the baseline study were included in the analyses. Only three recordings were disqualified on the basis of missing nighttime readings.

The characteristics of the study population at baseline are presented in Table 1. Those who died, were older, their BMI was higher, smoking was more frequent, and alcohol consumption was higher among them compared with those who survived. Coronary artery disease (CAD), diabetes, and previous stroke or TIA were more prevalent among those who died. Pulse pressure (PP) was higher, including office, 24‐h PP, daytime, and nighttime PP n those who did not survive compared with those who did. Office blood pressure was higher in those who deceased compared with the survivors. Also baseline ambulatory 24‐h mean systolic and diastolic BP, 24‐h daytime systolic and diastolic BP and nighttime systolic and diastolic BP blood pressure were all higher among those who died compared with those staying alive.

**Table 1:** Baseline characteristics of the study population by CV events and all‐cause mortality during follow‐up

	CV event n=208	No CV event n=692	*p* value	Deceased n=203	Alive n=697	*p* value	All n=900
**Females/Males (%)**	66 (31.7)/ 142 (68.3)	388 (56.1)/ 304 (43.9)	<.001	71 (35.0)/ 132 (65.0)	383 (54.9)/ 314 (45.1)	<.001	454 (49.6)/ 446 (50.4)
**Age (y)**	53.0 ± 5.7	50.9 ± 5.9	<.001	53.0 ± 5.8	50.9 ± 5.9	<.001	51.4 ± 5.9
**Body mass index (kg/m^2^)**	28.4 ± 4.3	27.4 ± 4.6	.006	28.5 ± 4.7	27.3 ± 4.5	.001	27.6 ± 4.6
**Smoking (%)**	67 (32.2)	188 (27.2)	.157	81 (39.9)	174 (25.0)	<.001	255 (28.3)
**Alcohol consumption (g/w)**	33 (3 – 122)	24 (2 – 72)	<.001	36 (3–120)	24 (2–72)	<.001	24 (2–84)
**CAD (%)**	45 (21.6)	57 (8.2)	<.001	31 (15.3)	43 (6.2)	<.001	74 (8.2)
**Hypertension (%)**	118 (56.7)	340 (49.1)	.055	112 (55.2)	346 (49.6)	.165	458 (50.9)
**Diabetes (%)**	35 (16.8)	56 (8.1)	<.001	37 (18.2)	54 (7.7)	<.001	91 (10.1)
**Stroke/TIA (%)**	9 (4.3)	8 (1.2)	.007	8 (3.9)	9 (1.3)	.015	17 (1.9)
**eGFR (mL/min/1.73 m^2^)**	84.8 ± 14.3	83.9 ± 15.0	.441	84.8 ± 15.7	83.9 ± 14.5	.443	84.1 ± 14.8
**Antihypertensive medication (%)**	126 (60.6)	334 (48.3)	.002	112 (55.2)	348 (49.9)	.188	460 (51.1)
**Office blood pressure (mmHg)**							
**Office SBP**	154 ± 22	147 ± 22	<.001	155 ± 24	146 ± 21	<.001	148 ± 22
**Office DBP**	92 ± 12	88 ± 12	<.001	92 ± 13	88 ± 12	<.001	89 ± 12
**Office PP**	62 ± 16	58 ± 15	.002	63 ± 16	58 ± 14	<.001	59 ± 15
**24‐h ABPM (mmHg)**							
**Mean SBP**	134 ± 15	129 ± 13	<.001	134 ± 15	129 ± 13	<.001	130 ± 14
**Mean DBP**	83 ± 9	80 ± 8	.001	82 ± 9	81 ± 8	.010	81 ± 8
**Daytime SBP**	139 ± 16	134 ± 13	<.001	139 ± 16	134 ± 13	<.001	135 ± 14
**Daytime DBP**	87 ± 9	85 ± 9	.002	87 ± 9	85 ± 9	.008	85 ± 9
**Nighttime SBP**	121 ± 15	116 ± 14	<.001	120 ± 16	116 ± 14	<.001	117 ± 14
**Nighttime DBP**	72 ± 9	70 ± 9	<.001	72 ± 10	70 ± 9	.023	70 ± 9
**Mean PP**	51 ± 10	48 ± 9	<.001	51 ± 10	48 ± 9	<.001	49 ± 9
**Daytime PP**	52 ±11	49 ± 9	<.001	52 ± 11	49 ± 9	<.001	50 ± 10
**Nighttime PP**	49 ± 10	46 ± 9	<.001	49 ± 10	46 ± 9	<.001	47 ± 9
**Fasting glucose (mmol/L)**	5.9 ± 1.2	5.6 ± 1.0	<.001	5.0 ± 1.6	4.6 ± 1.3	<.001	4.7 ± 1.4
**Fasting insulin (mU/L)**	12.3 (8.3 – 20.2)	10.1 (7.1 – 15.5)	<.001	12.6 (8.2 – 20.0)	10.2 (7.1–15.5)	<.001	10.6 (7.3 – 16.5)
**Total cholesterol (mmol/L)**	5.8 ± 1.2	5.7 ± 1.0	<.001	5.8 ± 1.2	5.7 ± 1.0	.069	5.7 ± 1.0
**HDL‐c (mmol/L)**	1.2 ± 0.4	1.4 ± 0.4	<.001	1.3 ± 0.4	1.4 ± 0.4	.025	1.3 ± 0.4
**LDL‐c (mmol/L)**	3.7 ± 1.0	3.5 ± 0.9	.001	3.6 ± 1.0	3.5 ± 0.9	.380	3.5 ± 0.9
**Triglycerides (mmol/L)**	1.5 ± (1.1 – 2.3)	1.2 ± (0.9 – 1.7)	<.001	1.5 ± (1.1 – 2.1)	1.2 ± (1.0 – 1.7)	<.001	1.31 (1.0 – 1.8)

Note: Data as mean ± SD, mean (25th–75th percentiles) or number (percentages). Statistical testing by ANOVA or chi‐square test between the groups.

Abbreviations: ABPM, ambulatory blood pressure measurement; CAD, coronary artery disease; DBP, diastolic blood pressure; eGFR, estimated glomerular filtration rate (CKD‐EPI); HDL‐c, HDL cholesterol; LDL‐c, LDL cholesterol; PP pulse pressure; SBP, systolic blood pressure;.

In laboratory measures, fasting glucose, insulin, and triglyceride levels were higher, and HDL cholesterol was lower among those who died, but there were no differences in total cholesterol or LDL levels.

During the follow‐up time of 21 years, 208 (23.1%) CV events (a major coronary incident, stroke, or cardiovascular death) occurred, and the majority (n=83, 39.9 %) of them in the highest 24‐h PP tertile. Men experienced 68.3 % of the CV events. Total number of deaths was 203 (22.6% of the study population). All‐cause mortality was the highest in the highest 24‐h PP tertile (30.5 % vs the lowest tertile 16.2%). All‐cause mortality among men (29.6%) was almost two‐fold compared with women (15.6%). There were 68 (33.5%, 51 men, 17 women) CV deaths, and 75% of those occurred in men.

Regarding CV events, those who experienced a CV event in comparison with those who were event‐free, the differences in above mentioned variables were quite similar (Table 1). However, total cholesterol and LDL levels were higher in CV event group, in which also antihypertensive medication was more frequent. Overall prevalence of use of antihypertensive agents in the whole study population was 51.1% (beta blocker 27.2%, ACE inhibitor 19.0%, thiazide 14.3%, calcium channel blocker 12.1%, loop diuretic 1.9%, and other agents 2.7%).

The baseline population was divided into tertiles according to 24‐h mean PP (Table 2). During the follow‐up time of 21 years, 208 (23.1%) CV events (a major coronary incident, stroke, or cardiovascular death) occurred, and the majority (n=83, 39.9 %) of them in the highest 24‐h PP tertile (Table 2). Men experienced 68.3% of the CV events. Total number of deaths was 203 (22.6% of the study population). All‐cause mortality was the highest in the highest 24‐h PP tertile (30.5 % vs the lowest tertile 16.2%. All‐cause mortality among men [29.6%] was almost two‐fold compared with women [15.6%]). There were 68 (33.5%, 51 men, 17 women) CV deaths, and 75% of those occurred in men.

**Table 2:** Baseline characteristics according to 24‐h pulse pressure tertiles

	1^st^ tertilen=309	2^nd^ tertilen=322	3^rd^ Tertilen=269	*p* value
Pulse pressure range	32 – 44	45 – 51	52 – 89	
Deceased (%)	50 (16.2)	71 (22.0)	82 (31.5)	<.001
CV event (%)	57 (18.4)	68 (21.1)	83 (30.9)	.001
Females (%)	149 (48.2%)	150 (46.6 %)	155 (57.6%)	.018
Age (y)	50.0 ± 5.6	51.0 ± 5.9	53.4 ± 5.9	<.001
Body mass index (kg/m^2^)	26.6 ± 4.0	27.6 ± 4.4	28.8 ± 5.1	<.001
Smoking (%)	79 (26 %)	95 (30 %)	81 (30%)	.406
Alcohol consumption (g/w)	30 (3 – 84)	24 (3 – 84)	17 (1 – 72)	.976
CAD (%)	21 (6.8)	23 (7.1)	30 (11.2)	.279
Hypertension (%)	136 (44.0)	158 (49.1)	164 (61.0)	<.001
Diabetes (%)	20 (6.5)	25 (7.8)	46 (17.1)	<.001
Stroke (%)	7 (2.3)	3 (0.9)	7 (2.6)	.225
eGFR (mL/min/1.73m^2^)	84 ± 15	85 ± 15	82 ± 15	.038
Antihypertensive medication (%)	138 (44.7)	160 (49.7)	162 (60.2)	.001
Office BP (mmHg)				
Office SBP	139 ± 19	146 ± 19	161 ± 22	<.001
Office DBP	89 ± 12	87 ± 12	91 ± 12	.001
Office PP	50 ± 11	59 ± 12	70 ± 15	<.001
24‐h ABPM				
Mean SBP	120 ± 8	128 ± 8	143 ± 13	<.001
Mean DBP	79 ± 7	80 ± 8	84 ± 9	<.001
Daytime SBP	125 ± 9	133 ± 9	148 ± 13	<.001
Daytime DBP	84 ± 7	84 ± 8	88 ± 10	<.001
Nighttime SBP	108 ± 10	115 ± 10	129 ± 14	<.001
Nighttime DBP	69 ± 9	69 ± 9	73 ± 10	<.001
Mean PP	40 ± 3	48 ± 2	60 ± 7	<.001
Daytime PP	41 ± 3	49 ± 2	61 ± 9	<.001
Nighttime PP	39 ± 4	45 ± 4	56 ± 10	<.001
Fasting glucose (mmol/L)	4.5 ± 0.9	4.6 ± 1.2	5.1 ± 1.9	<.001
Fasting insulin (mU/L)	9.5 (6.7 – 14.9)	10.8 (7.6 – 15.6)	11.9 (7.9 – 19.6)	<.001
Total cholesterol (mmol/L)	5.7 ± 1.0	5.7 ± 1.1	5.8 ± 1.0	.313
HDL‐c (mmol/L)	1.3 ± 0.4	1.4 ± 0.4	1.3 ± 0.4	.366
LDL‐c (mmol/L)	3.5 ± 0.9	3.5 ± 1.0	3.6 ± 0.9	.207
Triglycerides (mmol/L)	1.2 (0.9 – 1.7)	1.3 (1.0 – 1.8)	1.4 (1.1 – 1.9)	.001

Note: Data as mean ± SD, mean (25th–75th percentiles) or number (percentages). Statistical testing by ANOVA or chi‐square test between the groups.

Abbreviations: ABPM, ambulatory blood pressure measurement; BP, blood pressure; CAD, coronary artery disease; CV, cardiovascular; DBP, diastolic blood pressure; eGFR, estimated glomerular filtration rate (CKD‐EPI); HDL‐c, HDL cholesterol; LDL‐c, LDL cholesterol; PP, pulse pressure;; SBP, systolic blood pressure.

There were differences in incidence of CV mortality, CV events, and total mortality between the PP tertiles, and the linear associations were significant. All systolic and diastolic BP measurements, office, daytime and nighttime ambulatory, and pulse pressure, differed between the PP tertile groups. Prevalence of hypertension and diabetes increased by tertiles, but there were no statistically significant differences in prevalence of CAD nor previous stroke. In laboratory measurements, there were significant differences in fasting glucose, insulin, and triglycerides levels, and in estimated GFR between the tertiles. Prevalence of antihypertensive medication increased towards the highest PP tertile. There were statistically significant differences in thiazide and calcium blocker use, but not in other agents.

### 3.1 Pulse pressure and cardiovascular mortality

Pulse pressure as a predictor for CV mortality was analyzed with Cox regression by 24‐h, daytime, and nighttime ambulatory PP tertiles and by sex, as shown in Table 3. Adjustments were made for sex, age, BMI, diabetes, hypertension, antihypertensive medication use, CAD, previous stroke, alcohol consumption, smoking, triglycerides levels. High ambulatory 24‐h PP was independently associated with CV deaths in men (hazard ratio [HR] 2.98; confidence Interval [CI 95%] 1.11–8.04), but not in women (HR 1.18; 95% CI 0.16–8.95). Nighttime PP was a significant predictor of CV mortality in all (HR 2.60; 95% CI 1.08–6.3), but when sexes were analyzed separately, it appeared that nighttime PP was significant in men (HR 3.13; 95% CI 1.14–8.56), but not in women (HR 1.15; 95% CI 0.19–6.78). Daytime PP was of no statistical significance.

**Table 3:** Risk for cardiovascular mortality during 21 years of follow‐up by 24‐h pulse pressure tertiles

	**Males**		**Females**		**All**	
	HR (95% CI)	*p* value	HR (95% CI)	*p* value	HR (95% CI)	*p* value
**24‐h mean PP**						
**T1**		.080		.976		.488
**T2**	1.51 (0.63–3.56)	.355	1.22 (0.21–7.26)	.826	1.48 (0.71–3.08)	.295
**T3**	2.98 (1.11–8.04)	.031	1.18 (0.16–8.95)	.872	1.68 (0.68–4.10)	.259
**Daytime PP**						
**T1**		.284		.779		.560
**T2**	1.45 (0.62–3.37)	.391	0.99 (0.21–4.79)	.994	1.27 (0.62–2.60)	.522
**T3**	2.17 (0.83–5.69)	.115	0.56 (0.08–3.92)	.561	1.60 (0.68–3.77)	.282
**Nighttime PP**						
**T1**		.020		.987		.073
**T2**	3.37 (1.42–7.99)	.006	1.04 (0.22–5.02)	.957	2.18 (1.04–4.60)	.040
**T3**	3.13 (1.14–8.56)	.026	1.15 (0.19–6.78)	.880	2.60 (1.08–6.31)	.034

Cox regression models were adjusted for appropriate mean systolic pressure, age, body mass index, hypertension, diabetes, previous stroke, coronary artery disease, smoking, alcohol consumption, use of antihypertensive medication, triglycerides levels, and sex for analyses for all.

Abbreviations: CI, confidence interval; HR, hazard ratio; PP, pulse pressure; T, tertile.

*P* value <.05 considered as statistically significant.

### 3.2 Pulse pressure and cardiovascular events

We assessed the association of 24‐h, daytime, and nighttime ambulatory PP with CV events by multivariate Cox regression. The model was controlled by the same set of variables as in the Cox regression models presented before. When systolic mean ambulatory BP (24‐h, daytime, or nighttime, where applicable according the model) was added to Cox regression models, the statistical significance of high PP as a risk factor for CV events was lost in the whole study population (HR 1.12; 95% CI 0.69–1.84 for 24‐h PP, HR 1.45; 95% CI 0.89–2.35 for the daytime PP, and HR 1.33; 95% CI 0.82–2.14 for the nighttime PP). When the analyses were performed by sex, PP did not appear as a significant factor either. The result of the analyses is available as a supplemental material.

### 3.3 Pulse pressure and all‐cause mortality

**Figure 2:**
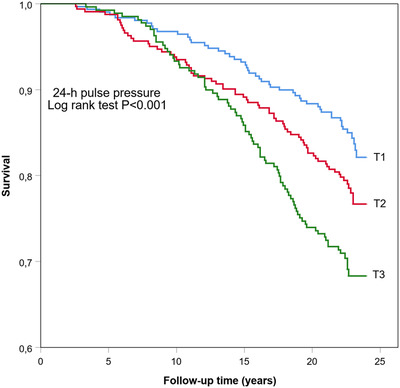
Kaplan–Meier all‐cause mortality survival curves by tertiles (T1, T2, T3) of 24‐h pulse pressure. T1, where PP ≤44 mmHg; T2, PP 45–51 mmHg; T3, PP >51 mmHg. Log Rank Test *p*<.001.

**Figure 3:**
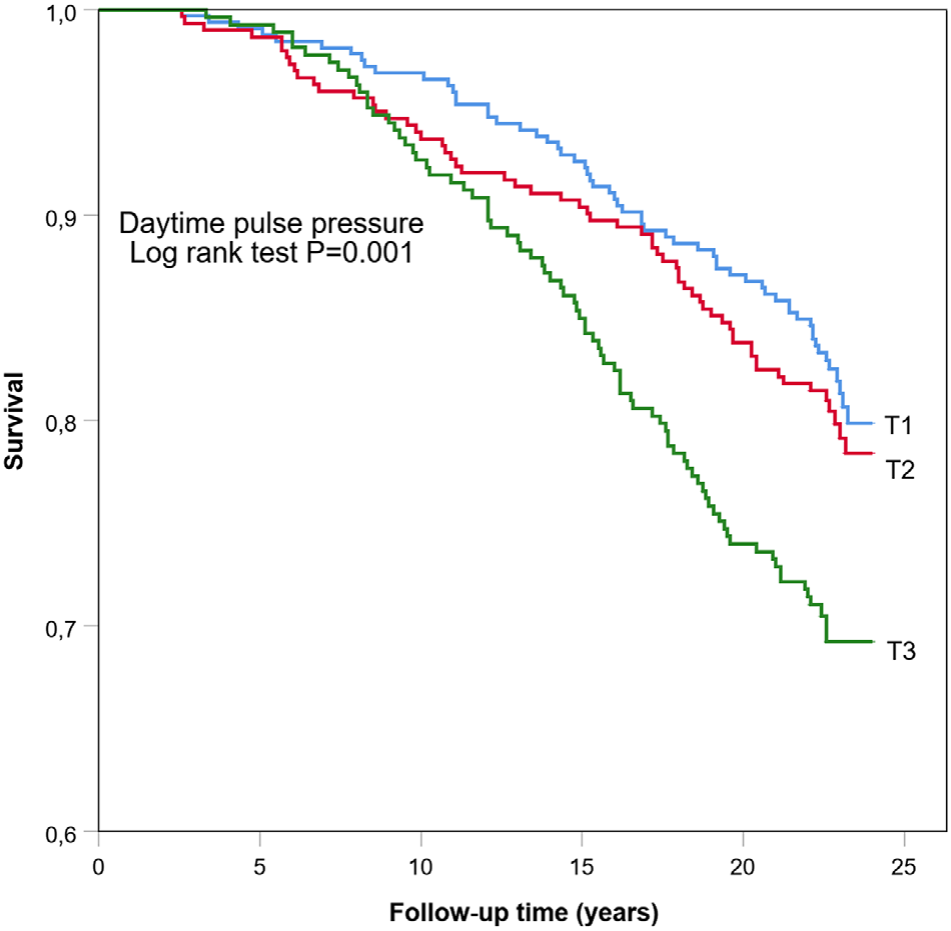
Kaplan–Meier all‐cause mortality survival curves by tertiles (T1, T2, T3) of daytime pulse pressure. T1, where PP ≤45 mmHg; T2, PP 46–52 mmHg; T3, PP >52 mmHg. Rank Test *p*=.001.

**Figure 4:**
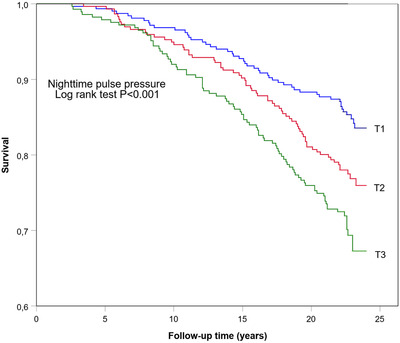
Kaplan–Meier all‐cause mortality survival curves by tertiles (T1, T2, T3) of nighttime pulse pressure. T1, where PP ≤42 mmHg; T2, PP 43–48 mmHg; T3, PP >48 mmHg. Log Rank Test *p*<.001.

Figures 2, 3, 4 show the cumulative Kaplan–Meier curves for hazards for all‐cause mortality in 24‐h, daytime, and nighttime PP tertiles. Ambulatory PP was assessed as a predictor for all‐cause mortality in multivariate Cox regression analyses separately for 24‐h, daytime, nighttime (Table 4), and for both sexes. Adjustments for sex, age, BMI, diabetes, hypertension, antihypertensive medication use, CAD, previous stroke, alcohol consumption, smoking, and triglycerides levels were made. The models also included ambulatory mean SBP—24‐h mean SBP for models assessing 24‐h PP, daytime mean SBP for models with daytime PP, and nighttime SPB for models evaluating nighttime PP, as appropriate to model. High nighttime PP was a significant independent predictor for all‐cause mortality in the whole population (HR 1.72, CI 95% 1.06–2.78) and separately in men (HR 2.26, 95% CI 1.29–3.96). High 24‐h PP predicted all‐cause mortality specifically in men (HR 2.40; CI 95% 1.32–4.37). Daytime PP, on the other hand, did not appear to be statistically significant.

**Table 4:** Risk for all‐cause mortality during 21 years of follow‐up by 24‐h pulse pressure tertiles

	**Males**		**Females**		**All**	
	HR (95% CI)	*p* value	HR (95% CI)	*p* value	HR (95% CI)	*p* value
**24‐h mean PP**						
**T1**		.009		.566		.456
**T2**	1.25 (0.76 ‐ 2.04)	.380	0.82 (0.45 ‐ 1.60)	.567	1.23 (0.83 ‐ 1.81)	.302
**T3**	2.40 (1.32 ‐ 4.37)	.004	0.62 (0.26 ‐ 1.49)	.287	1.36 (0.82 ‐ 2.26)	.230
**Daytime PP**						
**T1**		.329		.429		.843
**T2**	1.00 (0.62 ‐ 1.62)	.994	0.88 (0.45 ‐ 1.73)	.714	0.94 (0.64 ‐ 1.38)	.748
**T3**	1.44 (0.81 ‐ 2.57)	.217	0.57 (0.23 ‐ 1.41)	.224	1.05 (0.64 ‐ 1.72)	.838
**Nighttime PP**						
**T1**		.015		.621		.060
**T2**	1.68 (1.04 ‐ 2.71)	.034	1.20 (0.62 ‐ 2.29)	.592	1.49 (1.02‐ 2.19)	.042
**T3**	2.26 (1.29 ‐ 3.96)	.004	0.87 (0.37 ‐ 2.03)	.742	1.72 (1.06 ‐ 2.78)	.028

Cox regression models were adjusted for appropriate mean systolic pressure, age, body mass index, hypertension, diabetes, previous stroke, coronary artery disease, smoking, alcohol consumption, use of antihypertensive medication, triglycerides levels, and sex for analyses for all.

Abbreviations: CI, confidence interval; HR, hazard ratio; PP, pulse pressure; T, tertile.

*P* value < 0.05 considered as statistically significant.

## 4 DISCUSSION

In our 21‐year follow‐up study higher nighttime PP in all participants, and higher 24‐h PP in men were associated with the risk of CV and all‐cause mortality in a random cohort of middle‐aged population. The latter associations were evident after extensive adjustments including mean systolic ambulatory BP measurements.

The increasing of PP is a phenomenon usually seen with aging as SBP rises with age, while DBP begins to gradually decline after a plateau phase between 50 and 60 years.^1^ There is a sex difference,^27^ since women's earlier lower BP catches up with men's BP by the end of the sixth decade.^1^ The widening of PP is mostly due to reduced arterial elasticity, and PP is often considered as a surrogate measure for arterial stiffness. Our study participants were middle‐aged when investigated in the early 1990's—over 90% of them were under the age of 60, but still a significant **i**ndependent association between nighttime PP and CV as well as all‐cause mortality was detected.

Recently, Tadic and coworkers^17^ found out that 24‐h PP and daytime PP predicted CV events and mortality in a general population of wide age range, but nighttime PP was significant only in men. Earlier, 24‐h, daytime and nighttime PP were recognized as better prognostic factors for CV events in a group of 60 years of age or older, compared with a younger group.^28^ In a recent comparative outcome trial, with previously untreated elderly hypertensive patients, nighttime ambulatory PP was the most consistent pretreatment BP predictor of all‐cause and CV mortality during 11‐year follow‐up,^29^ which is in accordance with our 21‐year follow‐up study. Staessen and coworkers^12^ reported that high nighttime PP increased the risk of all‐cause and CV mortality in hypertensive patients over 60 years of age attending the placebo group. Antihypertensive medication was controlled by adjustments in the multivariate models in the present study. Compared with some earlier studies^28^ our investigation was characterized by generally lower PP levels, which may explain partly why 24‐h PP did not predict mortality or CV events in both genders.

In the studies by Khattar and Staessen^12^, ^28^ the association between high 24‐h PP and total mortality was also seen in the whole study group, whereas in the current OPERA study only among men. Arterial stiffness is a complex entity, which is especially affected by aging and sex hormones.^27^, ^30^ Decreasing levels of estrogen after menopause is likely to enhance arterial stiffening. In a large community‐based population, disappearance of the PP amplification between carotid and brachial arteries was a stronger predictor for all‐cause and CV mortality in women than in men, and more prominently in postmenopausal women.^31^ The majority of our female participants were presumably premenopausal or early menopausal in the beginning of the study. Therefore, it may be assumed that the arterial stiffness in women participants was lower than in younger women but still higher in average compared with the male study population of the same age. This may partly explain the gender difference, as none of the components of ambulatory PP were associated in CV events or mortality in women in the present study. The most probable explanation for this may be the fewer events and deaths among women compared with men, and the younger age range than above‐mentioned studies.^12^, ^28^

The reason why nighttime PP is an independent predictor for total mortality remains unknown. Several morbidities, such as hypertension and diabetes, may potentially increase PP and mortality. In our study, both hypertension and diabetes were associated with increased PP but controlling these diseases in the multivariate analysis did not change the results. In the present study the day‐ and nighttimes were fixed to certain hours and no data about participants sleeping habits were acquired. Another major concern is the lacking data on sleep apnoea, as sleep apnoea may affect the nighttime BP values.^32^ The practise of blood pressure treatment has developed towards combination medication since the beginning of our study, and the prevalence of renin‐angiotensin‐acting agents has increased substantially over the years. One limitation is also that study participants were caucasian only, therefore our results may not be applicable to other populations. However, the initial data with ABPM and a follow‐up of 21‐years is almost unique in the scientific world.

In conclusion, nighttime ambulatory pulse pressure showed a significant and independent association with CV and all‐cause mortality in a random cohort of middle‐aged normotensive and hypertensive participants in the long‐term follow‐up. Also 24‐h PP was detected as a prognostic factor in male participants. Wide PP, especially nighttime PP, may identify individuals with an increased risk for mortality, and therefore ambulatory BPM should be in use, together with office BP measurements.

## Supporting information

Supporting Information
