# Supplementary material for: Nighttime ambulatory pulse pressure predicts cardiovascular and all‐cause mortality among middle‐aged participants in the 21‐year follow‐up
Source: J Clin Hypertens (Greenwich). 2021 Jul 3;23(8):1547–55. doi: 10.1111/jch.14317 (PMC8678805; doi:10.1111/jch.14317)
Supplement: Supplementary file 1 — Supporting Information [file JCH-23-1547-s001.docx]

Supplementary table. Risk for cardiovascular events during 21 years of follow-up by pulse pressure tertiles

|  | Males | | Females | | All | |
| --- | --- | --- | --- | --- | --- | --- |
|  | HR (95% CI) | *P* value | HR (95% CI) | *P* value | HR (95% CI) | *P* value |
| 24-h mean PP |  |  |  |  |  |  |
| T1 |  | 0.406 |  | 0.836 |  | 0.861 |
| T2 | 0.94 (0.58 - 1.47) | 0.737 | 0.86 (0.41 - 1.79) | 0.687 | 1.01 (0.69 - 1.47) | 0.959 |
| T3 | 1.29 (0.72 - 2.29) | 0.389 | 1.06 (0.44 - 2.55) | 0.906 | 1.12 (0.69 - 1.84) | 0.641 |
| Daytime PP |  |  |  |  |  |  |
| T1 |  | 0.611 |  | 0.788 |  | 0.325 |
| T2 | 1.18 (0.75 - 1.87) | 0.472 | 1.30 (0.61 - 2.78) | 0.493 | 1.18 (0.80 - 1.73) | 0.402 |
| T3 | 1.32 (0.75 - 2.33) | 0.330 | 1.21 (0.48 - 3.08) | 0.688 | 1.45 (0.89 - 2.35) | 0.135 |
| Nighttime PP |  |  |  |  |  |  |
| T1 |  | 0.561 |  | 0.011 |  | 0.387 |
| T2 | 1.23 (0.80 - 1.91) | 0.347 | 1.94 (0.94 - 3.99) | 0.071 | 1.28 (0.88- 1.87) | 0.194 |
| T3 | 1.03 (0.59 - 1.79) | 0.918 | 0.76 (0.28 - 2.03) | 0.585 | 1.33 (0.82 - 2.14) | 0.351 |

Cox regression models were adjusted for appropriate mean systolic pressure, age, body mass index, hypertension, diabetes, previous stroke, coronary artery disease, smoking, alcohol consumption, use of antihypertensive medication, triglycerides levels, and sex for analyses for all.

Abbreviations: HR, hazard ratio; CI, confidence interval; PP, pulse pressure; T, tertile.

*P* value < 0.05 considered as statistically significant.
